# A new wide input voltage DC-DC converter for solar PV systems with hybrid MPPT controller

**DOI:** 10.1038/s41598-024-61367-x

**Published:** 2024-05-09

**Authors:** Sunkara Sunil. Kumar, K. Balakrishna

**Affiliations:** Vignan’s Foundation for Science Technology and Research, Vadlamudi, India

**Keywords:** Duty cycle, Efficiency of the converter, Fast MPP tracking speed, Good system dynamic response, And high robustness, Engineering, Electrical and electronic engineering

## Abstract

The present working conventional power generation systems utilization is reducing day by day because of their demerits are more functioning cost, high carbon dioxide emission, more complexity in handling, and required high installation area. So, the current power generation company focuses on Renewable Energy Sources (RES) which are wind, tidal, and solar. Here, the solar power network is utilized for supplying electricity to the electrical vehicle battery charging system. The Solar photovoltaic (PV) modules supply nonlinear power which is not useful for automotive systems. To maximize the supply power of the solar PV system, an Adaptive Step Genetic Algorithm Optimized (ASGAO) Radial Basis Functional Network (RBFN) is utilized for tracking the working point of the solar PV module thereby enhancing the operating efficiency of the overall system. The features of this proposed hybrid Maximum Power Point Tracking (MPPT) controller are quick system dynamic response, easy operation, quick convergence speed, more robustness, and high operating efficiency when equalized with the basic MPPT controllers. The major issue of solar PV modules is low supply voltage which is increased by introducing the wide input voltage DC-DC converter. The merits of this introduced converter are low-level voltage stress on diodes, good quality supply power, high voltage gain, plus low implementation cost. Here, the introduced converter along with the AGAO-RBFN controller is analyzed by selecting the MATLAB/Simulink environment. Also, the proposed converter is tested with the help of a programable DC source.

## Introduction

As of now, the researchers and power generation industries are highly focused on Renewable Energy Sources (RES) because of their high flexibility, ease of maintenance, less human resources needed, and high adaptability when competing with conventional power networks. From the literature review, the conventional power networks are named natural gas, oil, nuclear, tar sand, crude oil, phosphate, and coal. In a natural gas power network, the natural gas is added with the water steam to operate the gas turbine with constant velocity^1^. Here, the waste heat comes out from the gas turbine which is recycled for processing the natural gas. The natural gas power system applications are room heating, heavy-duty transportation, air conditioning systems, and cooking applications. The merits of this system are relatively low functioning cost and less hydrocarbon emissions.

However, the drawback of this system is not suitable for large-scale load applications. So, the nuclear system is focused on^2^ for limiting the demerits of natural gas power supply networks. Here, the nuclear energy is harvested by splitting the uranium material into small pieces of atoms which is called as fission process. At the time of uranium clustering, there is a huge amount of heat is produced which helps to produce the heated steam. The available heated steam is forcibly sent to the steam turbine chamber for functioning the electric generator^3^. The foremost features of nuclear systems are very low carbon emissions in the atmosphere, high energy density, easy transportation of uranium material, and superior reliability. However, the uranium-related power networks' drawbacks are expensive starting costs to develop, high risk of accidents, more radioactive wastage, limited fuel availability, and moderate impact on nature^4^. Also, its radioactive material explosions create many issues for human beings. The coal power plants are installed in India to limit the disadvantages of uranium-based power networks.

In this coal power system, initially, the coal is collected from the coalmines. After that, the collected coal is transferred to the boiler chamber for producing the steam. The high-pressurized steam is utilized for running the rotor of the machine. The rotating generator supplies the electricity to the distribution system^5^. In^6^, the authors reviewed the various types of thermal power networks for supplying electricity to the automotive industry. Based on the literature study, the authors concluded that the thermal power networks supply a huge amount of power to the peak load conditions. Here, the extraction of peak power from the thermal system is easy, and the purchasing cost of the coal is much less. Also, it needed less catchment area for the installation of thermal power stations when compared to the hydropower stations^7^. However, the rotating turbine cost of the thermal power station is very high. In addition, the coal power supply station emits a huge amount of smoke and pollutants in the atmosphere. The functioning efficiency of this system is very poor. So, the present power supply industries going towards renewable energy networks which are illustrated as hydropower, wind, geothermal, solar, and tidal electrical networks^8^.

The hydropower stations are installed near the water storage places. Here, the high-head water kinetic power is converted into the mechanical power source for running the generator. Hydropower is a clean source of energy, and it is a domestic source of energy. In addition, it is highly flexible and reduces the risk of flooding. However, the construction of dams creates a high atmospheric impact^9^. So, in this work, the solar power station is selected for supplying power to the automotive systems. Solar is the most important and freely available energy source and it is a nonmechanical device that absorbs the sunlight irradiation energy and transfers it to useful electrical power. From the previously available manuscripts, solar cells are being developed by utilizing various materials which are polysilicon, ingot, and wafer^10^. The thin film PV system is manufactured by selecting the cadmium telluride along with the float gas. The float gas is fully coated with the transparent conductive layer. In addition to this, the solar PV modules are implemented by utilizing polycrystalline, plus monocrystalline methodologies. However, the unique solar cell power is not useful for local consumers. So, the PV module manufacturers utilize a multiple number of cells to form an array. As a result, the PV system power supply capability is enhanced^11^.

**Figure 1:**
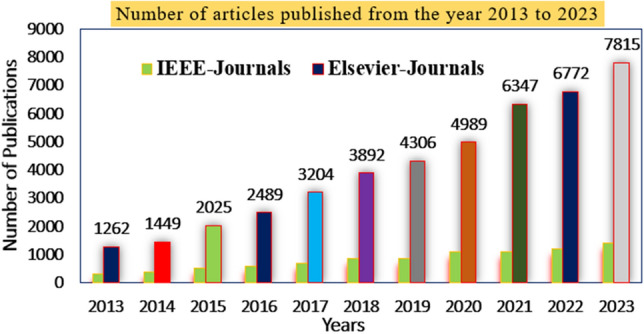
Present importance of MPPT techniques for RESs,^13^.

All the solar power networks supply continuously fluctuated output power because of the quick changes in the sunlight intensity. Also, it produces nonlinear voltage which is not acceptable for the automotive industry. So, various researchers working on the new development of MPPT methodologies which are separated as artificial intelligence, nature-inspired optimization, conventional methodologies, neural computing techniques, and evolutionary controllers, and its publication status is provided in Fig. 1. In the paper^12^, the authors utilized the P&O concept for smart grid power distribution to rural areas peoples. In this controller block, the solar power is changed continuously towards the Maximum Power Point (MPP) of the P-I curve of the solar system. The major merits of this controller are flexible design, good stability for uniform irradiation conditions of the solar system, and less development cost, plus few iterations are employed to find out the best optimal solution. However, it is applicable where the accuracy of the system is not necessary. Also, this controller gives more distortions in the system voltage^13^. The MIC methodology is interfaced in the wind/PV hybrid system for supplying the switching pulses to the double bridge DC-DC converter circuit thereby improving the stability of the EV load voltage. The development cost of this method is a little high when equated with the P&O method.

The slider technology is applied to the solar power interfaced battery charging network for charging the battery to run the automotive system with high efficiency. In this system, the slider continuously monitors the solar movement thereby tracking the functioning point of the solar PV system^14^. The merits of this controller are fast maximum power point tracking speed, more flexibility under various sunlight intensity conditions, easy handling capability, plus highly suitable for the rapid variation of natural conditions. However, the sliders provide less efficiency for highly complex systems. Also, it does not accept the slow convergence rate of the solar MPP. Most of the smart grid networks produce more distorted voltages which are useful at the time of solar MPP finding in the ripple correlation method. This method of design is very easy because it doesn’t require many more components for the development of a ripple correlation-based sunlight system. Here, by using the ripple correlation block, the proposed system implementation price is improved which is not acceptable for domestic consumers^15^.

In the article^16^, the manufacturers used the state space model for supplying the duty pulses to the multiple input sources based interleaved converter for enhancing the efficiency of the fuel stack system. Here, all the state variables of the fuel stack, and converter are selected for tracking the working point of the fuel stack. The merits of this controller are high accuracy in tracking the functioning point of the overall system, ease of handling the controller, low level of system complexity, and more reliability. The major issue of the state space model is highly computationally intensive. The linearized feedback loop is utilized in the PV/fuel stack/battery system for controlling the state of charge of the electric vehicle system. In this feedback controller, all the source's load line slope is evaluated for controlling the all-quadratic power converter circuits. The drawback of this methodology is only suitable for uniform solar temperature conditions^17^.

**Figure 2:**
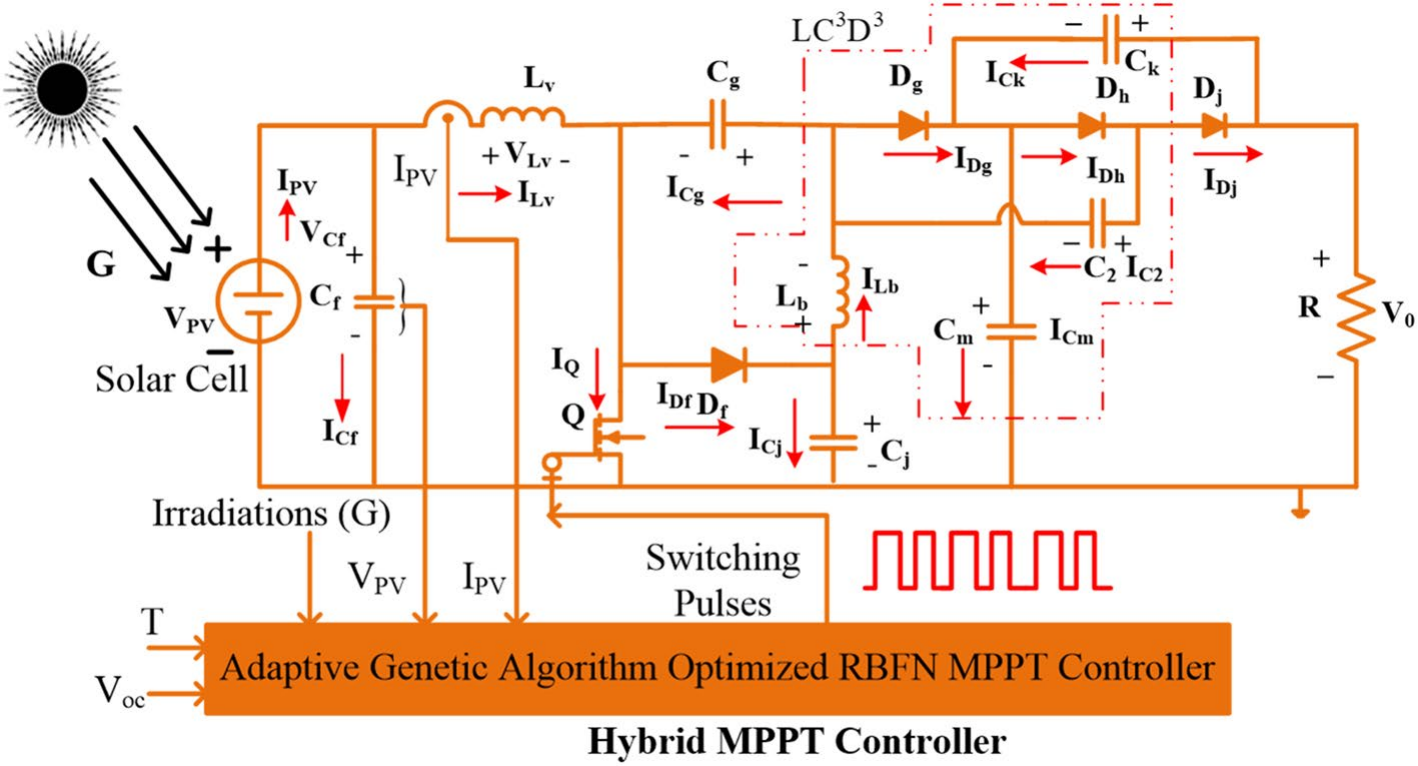
Proposed universal supply voltage DC-DC converter with RBFN MPPT controller.

So, the drawbacks of conventional methodologies are limited by the GA-optimized Adaptive Adjustable Step-Radial Basis Functional Network concept proposed in this work to track the accurate position of the solar MPP. Here, From Fig. 2, the collected signals to the proposed MPPT block are Irradiations (G), PV supplied voltage (V_PV_), sunlight temperature (T), Open Circuited Voltage (V_0C_), plus converter collected current (I_PV_). Most of the PV module's installation price is very high which is compensated by utilizing the different categories of power converter topologies which are wireless, interleaved, quadratic, and non-isolated converters. In^18^, the researchers worked on isolated technology-related converters which are forward power converters, plus fly-back converters. In this type of converter, there is a separation made by using the transformer between the supply, and load. This transformer in the converter protects the diodes, and MOSFETs from the sudden variation in supply voltages. However, this type of circuit occupies more space for installation, requires more electrical elements for developing the isolated-based converter, more power distribution losses, low operational efficiency, plus more temperature sensitivity^19^.

So, the present electric vehicle battery charging networks are focusing on the quadratic transformerless universal supply voltage DC-DC converter circuits for optimizing the overall system size, plus design complexity^20^. General transformerless-related power converters are Luo, Buck-Boost, Cuk, zeta, and SEPIC converters. These power converters are utilized for moderate voltage gain applications. Here, in this article, a single switch, wide voltage gain, uniform supply voltage DC-DC converter is introduced for boosting the voltage of the solar system. The features of the proposed single-switch converter are low-level voltage distortions in the system, more voltage conversion ratio, less voltage stress on thIMPLEMENTATION OF GA-RBFN BASED MPPT CONTROLLERe diodes, plus less component utilization.

So, the overall proposed network implementation cost is reduced. The remaining part of the article is followed as the design, plus partial shading conditions of the solar system are explained in “Implementation of triple diode solar cell”, and “Shading phenomena of solar PV systems”. A detailed analysis of various MPPT controllers is given in “Implementation of GA‑RBFN based MIPPT controller”. The development of the converter and analysis of various MPPT controllers are given in “Development of universal input supply DC‑Dc converter”. The analysis and experimental testing of the converter are provided in “Simulation results” and “Experimental verification of the proposed USHVBC”. Finally, in “Conclusion”, the conclusion of the article is provided.

## Implementation of triple diode solar cell

**Figure 3:**
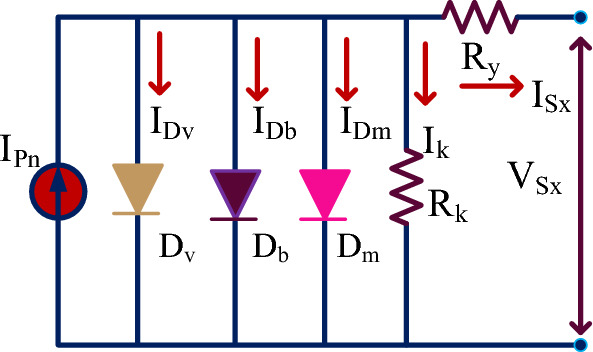
Utilized 3-diode solar cell for developing the PV array.

The solar cells are manufactured by selecting the silicon material. The PV cell formation has been done by combining the two types of charges which are N-type charges, plus P-type charges^21^. Here, the N-type device is highly filled with electrons, and the P-type device is heavily filled with holes. So, the functioning of the solar system is depending on the P-N device operation. Most of the review articles say that the single diode, plus double diode cells are the approximated models. As a result, it may not give the accurate results of the sunlight systems. So, in this work, a 3-diode type solar cell is proposed and its parameters are selected by using the wind-driven optimization algorithm. The selected triple diode structure is given in Fig. 3. From Fig. 3, the major utilized parameters for the implementation of this solar cell are ideality factors, peak power of the cell, open-circuited voltage, peak voltage of the cell, plus short-circuited currents. Here, when the shunt resistance of the 3-diode solar cell is removed then the PV cell current is evaluated by using Eq. (1). Suppose, the shunt resistive element is included in the circuit then the PV generated current is determined by using the Eq. (4).

1 $${{\text{I}}}_{{\text{Sx}}}={{\text{I}}}_{{\text{Pn}}}-{{\text{I}}}_{{{\text{D}}}_{{\text{v}}}}-{{\text{I}}}_{{{\text{D}}}_{{\text{b}}}}-{{\text{I}}}_{{{\text{D}}}_{{\text{m}}}}$$

2 $${{\text{I}}}_{{\text{Sx}}}={{\text{I}}}_{{\text{Pn}}}-{{\text{I}}}_{{\text{or}}\_{\text{v}}}\left({{\text{e}}}^{\frac{{\text{q}}*({{\text{V}}}_{{\text{Sx}}}+{{\text{I}}}_{{\text{Sx}}}{{\text{R}}}_{{\text{y}}})}{{\upeta }_{{\text{v}}}*{\text{K}}*{\text{T}}}}-1\right)-{{\text{I}}}_{{\text{z}}}$$

3 $${{\text{I}}}_{{\text{z}}}={{\text{I}}}_{{\text{or}}\_{\text{b}}}\left({{\text{e}}}^{\frac{\left({{\text{V}}}_{{\text{Sx}}}+{{\text{I}}}_{{\text{Sx}}}{{\text{R}}}_{{\text{y}}}\right)*{\text{q}}}{{\upeta }_{{\text{b}}}{\text{KT}}}}-1\right)+ {{\text{I}}}_{{\text{or}}\_{\text{m}}}\left({{\text{e}}}^{\frac{\left({{\text{V}}}_{{\text{Sx}}}+{{\text{I}}}_{{\text{Sx}}}{{\text{R}}}_{{\text{y}}}\right)*{\text{q}}}{{\upeta }_{{\text{m}}}{\text{KT}}}}-1\right)$$

4 $${{\text{I}}}_{{\text{Sx}}}={{\text{I}}}_{{\text{Pn}}}-{{\text{I}}}_{{\text{Dv}}}-{{\text{I}}}_{{\text{Db}}}-{{\text{I}}}_{{\text{Dm}}}-{{\text{I}}}_{{\text{k}}}$$

5 $${{\text{I}}}_{{\text{Sx}}}={{\text{I}}}_{{\text{Pn}}}-{{\text{I}}}_{{{\text{or}}\_}_{{\text{V}}}}\left({{\text{e}}}^{\frac{{\text{q}}*({{\text{V}}}_{{\text{Sx}}}+{{\text{I}}}_{{\text{Sx}}}{{\text{R}}}_{{\text{y}}})}{{\upeta }_{{\text{v}}}*{\text{K}}*{\text{T}}}}-1\right)- {{\text{I}}}_{{\text{or}}\_{\text{b}}}\left({{\text{e}}}^{\frac{\left({{\text{V}}}_{{\text{Sx}}}+{{\text{I}}}_{{\text{Sx}}}{{\text{R}}}_{{\text{y}}}\right)*{\text{q}}}{{\upeta }_{{\text{b}}}{\text{KT}}}}-1\right)-{{\text{I}}}_{{\text{q}}}$$

6 $${{\text{I}}}_{{\text{q}}}={{\text{I}}}_{{\text{or}}\_{\text{b}}}\left({{\text{e}}}^{\frac{\left({{\text{V}}}_{{\text{Sx}}}+{{\text{I}}}_{{\text{Sx}}}{{\text{R}}}_{{\text{y}}}\right)*{\text{q}}}{{\upeta }_{{\text{b}}}{\text{KT}}}}-1\right)+\frac{{{\text{V}}}_{{\text{Sx}}}+{{\text{I}}}_{{\text{Sx}}}{{\text{R}}}_{{\text{y}}}}{{{\text{R}}}_{{\text{k}}}}$$

7 $${{\text{I}}}_{{\text{or}}\_{\text{v}}}={{\text{I}}}_{{\text{or}}\_{\text{b}}}={{\text{I}}}_{{\text{or}}\_{\text{m}}}={{\text{I}}}_{{\text{nm}}}{{\text{e}}}^{\frac{{\text{qEg}}}{{\text{nk}}}\left(\frac{1}{{{\text{T}}}_{{\text{N}}}}-\frac{1}{{\text{T}}}\right)}*(\frac{{\text{T}}}{{{\text{T}}}_{{\text{N}}}}{)}^{3}$$

8 $${{\text{I}}}_{{\text{nm}}}={{\text{I}}}_{{\text{nm}}}={{\text{I}}}_{{\text{nm}}}={{\text{I}}}_{{\text{nm}}}=\frac{{{\text{I}}}_{{\text{SC}}}}{{{\text{e}}}^{\left(\frac{{{\text{V}}}_{{\text{oc}}\_{\text{n}}}}{\upeta {{\text{V}}}_{{\text{Tn}}}}\right)}}$$

## Shading phenomena of solar PV systems

Most of the solar systems are located near to the shadow free region. From the literature study, the present solar systems are located on the rooftop^22^. So, the building shadow creates the discontinuity in the power supply of the solar PV systems. In addition, tree falling, plus cloudy conditions create the shading effect on the PV modules thereby reducing the functioning efficiency of the solar systems. Due to this shading effect, the solar systems supply highly fluctuated nonlinear multiple peaks voltage versus current characteristics^23^. The effect of solar module shading is mentioned in Fig. 2. From Fig. 2, the shading reduces the power generation capability of the solar PV systems. So, a diode is placed across each PV module to remove the reverse leakage currents of the overall proposed system. Also, these diodes reduce the power consumption of the shaded solar PV modules.

**Figure 4:**
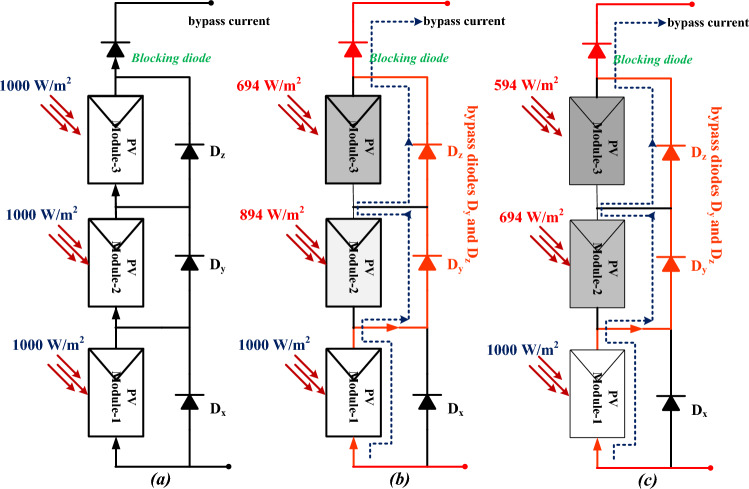
(**a**). Collection of uniform irradiation values, (**a**). Shading condition-1, plus (**b**). Shading Conditions-2.

**Figure 5:**
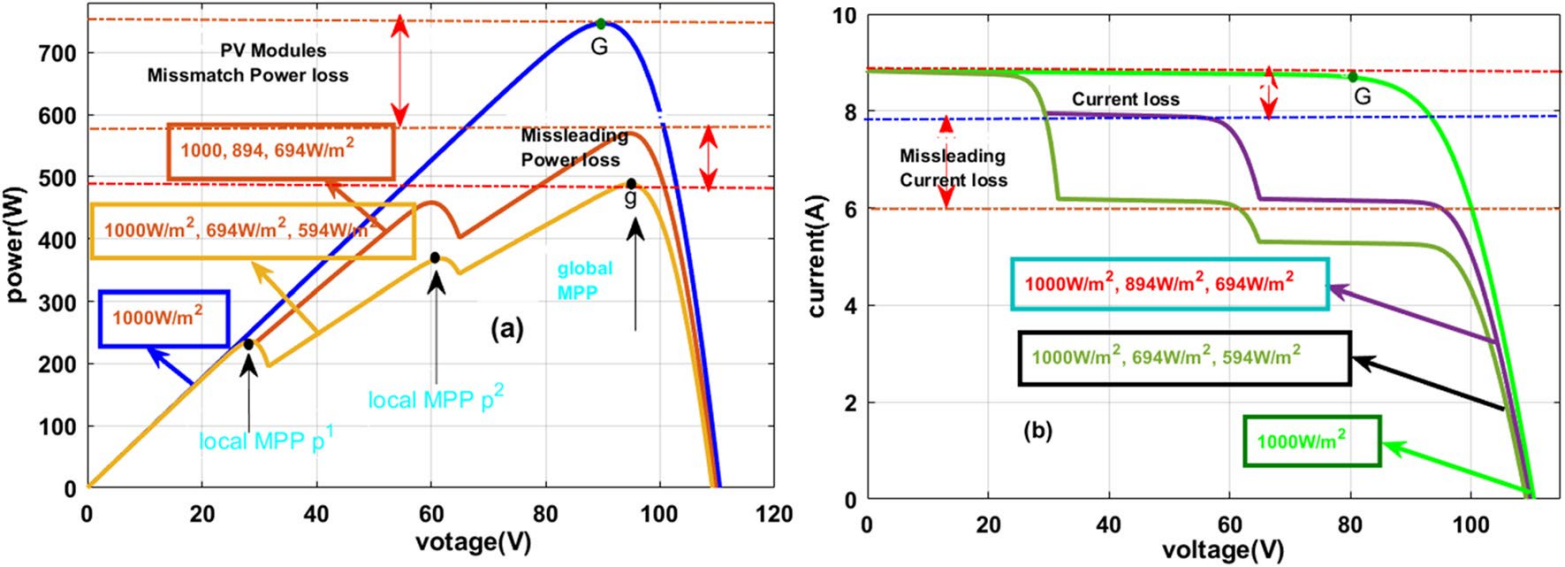
(**a**) Sunlight PV system P–V curves, plus (**b**) Sunlight PV system I-V curves.

Here, there are three solar modules are utilized for the analysis of partial shading conditions of the solar PV system. Here, one solar string is considered as uniform irradiation condition which is mentioned in Fig. 4a, and the remaining two are selected as the partially shaded solar PV modules as given in Fig. 4b, and Fig. 4c. From Fig. 4a, all three PV modules receive the 1000W/m^2^ irradiations and in the 1^st^ shading condition, the collected irradiations from the sunlight are 1000W/m^2^, 894W/m^2^, and 694W/m^2^. Finally, the utilized irradiation values for the 3^rd^ shading condition are 1000W/m^2^, 694W/m^2^, and 594W/m^2^. The generated voltage versus current characteristics under shaded and uniform irradiations of the solar PV system are illustrated in Fig. 5a, and Fig. 5b.

From Fig. 5a, all the 3-modules generated power (P_MPP_) is 739.9W, and its related maximum voltage (V_MPP_) is 94.21W. As usual, the generated powers and voltages at the first and 2nd shading conditions of the solar system are 581.55W, 498.88W, 92.08 V, and 90.8 V respectively. From Fig. 5b, at constant sunlight irradiation values, the functioning point of the PV system is one. There are three peak power points at rapid changes of the sunlight intensity conditions which are named p^1^, p^2^, and g. Here, p^1^, plus p^2^ are the normal peak power points, and ^‘^g^’^ is represented as the global maxima point. Due to this 1st and 2nd shading effect on the solar PV systems, the mismatch and misleading power losses of the overall system are 159.95W, and 84.21W respectively.

## Implementation of GA-RBFN based MIPPT controller

The solar system's nonlinear performance disturbs the overall system efficiency which leads to high power losses in the converter circuit^24^. So, the MPPT controller plays a major predominant role in the all-renewable energy systems in order to extract the peak power from the PV network. Also, the MPPT block in the renewable energy system moves the functioning point of the PV network near to the real MPP position. This MPPT controller helps the solar system to meet the consumer load demand. Based on this MPPT importance, here, in this manuscript, the GA-RBFN methodology is implemented to overcome the shading issue of solar networks. Along with the GA-RBFN, the recent other MPPT methodologies are investigated which are P&O-ANN, ANN-HC, and GA with P&O controllers.

### P&O-based Artificial Intelligence MPPT controller

**Figure 6:**
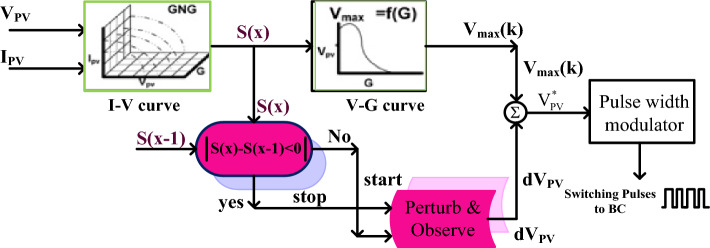
Schematic representation of P&O-ANN MPPT controller for PV system.

The conventional methodologies' major problems are the highest oscillations across the PV MPP, discontinuity in the consumer utilization power, unable to track the exact MPP location, more power distribution losses, plus less peak power extraction from the sunlight system^25^. So, the P&O methodology is applied to the shaded solar system to run the operating point of the overall system from the origin to the actual functioning point. Once the functioning point meets the original MPP location then the artificial intelligence concept is utilized in the MPPT block as shown in Fig. 6. From Fig. 6, the ANN-generated error output signal is feedback to the P&O block to obtain the peak voltage of the PV. The evaluated peak voltage is added with the reference solar voltage for adjusting the duty signal of the proposed DC-DC power converter circuit. The features of this P&O-ANN controller in the proposed system are more accurate output power generation, moderate complexity in understanding, plus easy design and development. However, this controller is less flexible for rapid changes in sunlight temperature conditions.

### ANN with hill climb MPPT controller

**Figure 7:**
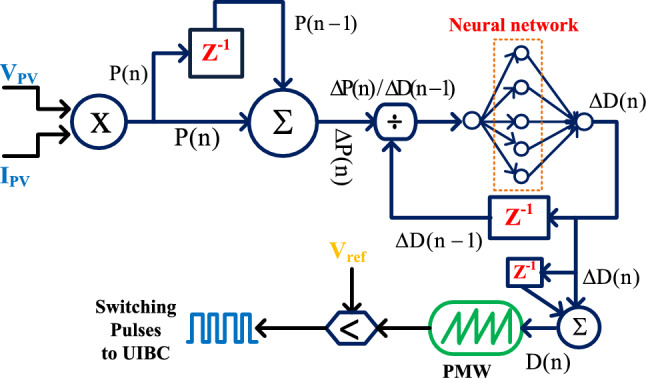
Switching pulses creation by exploiting the ANN-HC.

From the previously published articles, the hill climb methodology provides very low solar power output in the smart grid power supply network^26^. Also, it takes high development cost when equated with the basic P&O methodology. So, the limitations of the hill climb methodology are compensated by combining the artificial intelligence block thereby handling the shading condition of the solar system. In this hybridization, the drawbacks of ANN and HC are neglected to improve the stability of the microgrid system. The overall working structure of this hybrid controller is represented in Fig. 7. From Fig. 7, the solar network produced voltage, plus currents are sent to the artificial neural network block for identifying the error peak power of the sunlight system.

### GA with P&O power point tracking controller

**Figure 8:**
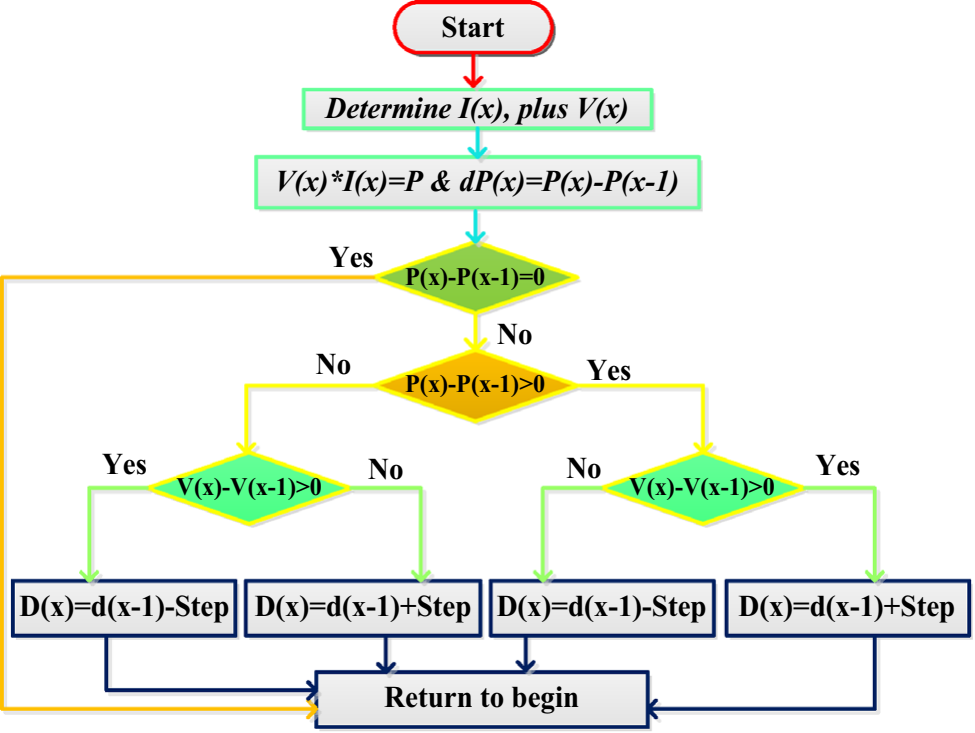
Genetic Algorithm based P&O MPPT controller.

In the article^27^, the reviewers studied the genetic algorithm for PV/wind power distribution system to stabilize the power of local consumers. Here, there are twenty-four agents are considered and which are initiated with duty values in between the range of 0.15 to 0.99. From these duty values, the GA tries to move the operating point of the hybrid PV/wind power supply network from the local peak power point position to the global peak power point place. Once, the solar peak power point crosses the local MPPs then the P&O block takes the solar network voltage, plus power for continuous adjustment of the period of the switching signals as shown in Fig. 8. From Fig. 8, when the working point of the sunlight system is either left side corner or right-side corner of the P–V curve then Eq. (9) is applied to the overall system to supply peak power to the electric vehicle network.

9 $${\text{D}}({\text{x}})={\text{D}}({\text{x}}-1)\pm {\text{u}}*\left(\frac{{\text{p}}({\text{x}})-{\text{p}}({\text{x}}-1)}{{\text{v}}({\text{x}})-{\text{v}}({\text{x}}-1)}\right)$$

10 $${\text{Step}}\,{ = }\,{\text{u}}\left( {\tfrac{{\text{p(x) - p(x - 1)}}}{{\text{v(x) - (x - 1)}}}} \right)$$

where the constraints D(x), and P(x) are the present available duty value of the power converter, and load power. Finally, the terms D(x-1), plus P(x-1) are stored duty signal value, and converter power value.

### Proposed adjustable Size GA-RBFN hybrid controller

From the previously published literature study, the limitations of this genetic controller are premature convergence, slow MPP tracking speed, plus needed parameters tunning. Similarly, artificial neural networks have the drawbacks of proneness to overfitting, plus the empirical nature of system development. Also, these neural networks consume more energy and create more ethical issues^28^. So, the ANN is included in the GA block to enhance the system convergence speed, improve the MPP tracking accuracy, more adaptability for quick changes in environmental conditions, better dynamic system response, plus provide more stable output power to the consumer.

**Figure 9:**
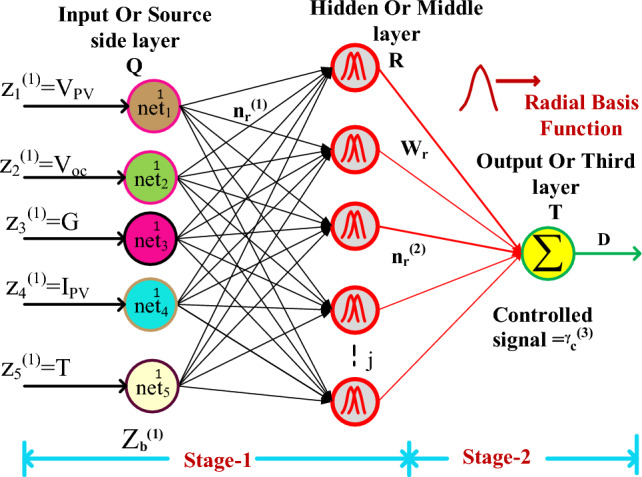
Introduced GA-RBFN Power Point Tracking Controller.

In this MPPT controller strategy, the GA optimization updates the neuron weights until the solar system reaches the MPP position. After that, the RBFN circuit is interfaced in the solar system to obtain the duty value to the universal supply voltage DC-DC converter. The generation of duty signal to the introduced DC-DC converter is illustrated in Fig. 9. From Fig. 9, the utilized parameters for obtaining the duty of the converter are peak solar voltage, irradiations of the sunlight, open circuit voltage of the overall system, peak solar current, plus functioning temperature of the sunlight. In this proposed controller, there are two methodologies are applied for initializing the neuron's weights in the RBFN circuit and adjusting their corresponding weights. Here, the source side neural network layer weights are monitored by selecting the supervised learning, and the middle layer radial basis function training has been performed by applying the unsupervised learning.

11 $${{\text{n}}}_{{\text{q}}}^{1}({\text{j}})={{\text{f}}}_{{\text{q}}}^{1}{{\text{net}}}_{{\text{q}}}^{1}({\text{j}})={{\text{net}}}_{{\text{q}}}^{1}({\text{j}});{\text{j}}=\mathrm{1,2}..{\text{m}}$$

12 $${{\text{net}}}_{{\text{r}}}^{2}({\text{j}})=-({\text{x}}-{{\text{m}}}_{{\text{r}}}{)}^{{\text{T}}}{\sum }_{{\text{r}}}({\text{x}}-{{\text{m}}}_{{\text{r}}});{\text{j}}=1.2,{\text{m}}$$

13 $${{\text{n}}}_{{\text{r}}}^{2}({\text{j}})={{\text{f}}}_{{\text{r}}}^{2}{{\text{net}}}_{{\text{r}}}^{2}({\text{j}})={{\text{net}}}_{{\text{r}}}^{2}({\text{j}});{\text{j}}=1.2..,{\text{m}}$$

14 $${{\text{net}}}_{{\text{t}}}^{3}={\sum }_{{\text{r}}}{{\text{W}}}_{{\text{r}}}*{{\text{n}}}_{{\text{t}}}^{2}({\text{j}});{\text{j}}=1.2.....,{\text{m}}$$

15 $${n}_{{\text{t}}}^{3}\left({\text{j}}\right)={{\text{f}}}_{{\text{t}}}^{3}{{\text{net}}}_{{\text{t}}}^{3}\left({\text{j}}\right)={{\text{net}}}_{{\text{t}}}^{3}\left({\text{j}}\right);{\text{j}}=1.2\dots ,{\text{m}}$$

16 $${\text{error}}={\sum }_{{\text{j}}=1}^{{\text{z}}}\frac{1}{2}\left({{\text{D}}}_{{\text{ref}}}-{{\text{D}}}_{{\text{actual}}}\right)$$

From Eq. (11), the term ${{\text{n}}}_{{\text{q}}}^{1}({\text{j}})$ represents the supply layer net value, q, and j are denoted as layer number, and the total number of nodes in the supply layer. Similarly, Q, R, and T are the identification of layers in the ANN. Here, x, and m are the mean values of 1st and 2nd layers. Finally, the variable ${{\text{n}}}_{{\text{t}}}^{3}\left({\text{j}}\right)$ represents the load layer net value which helps supply the switching signals to the power converter.

## Development of universal input supply DC-Dc converter

**Figure 10:**
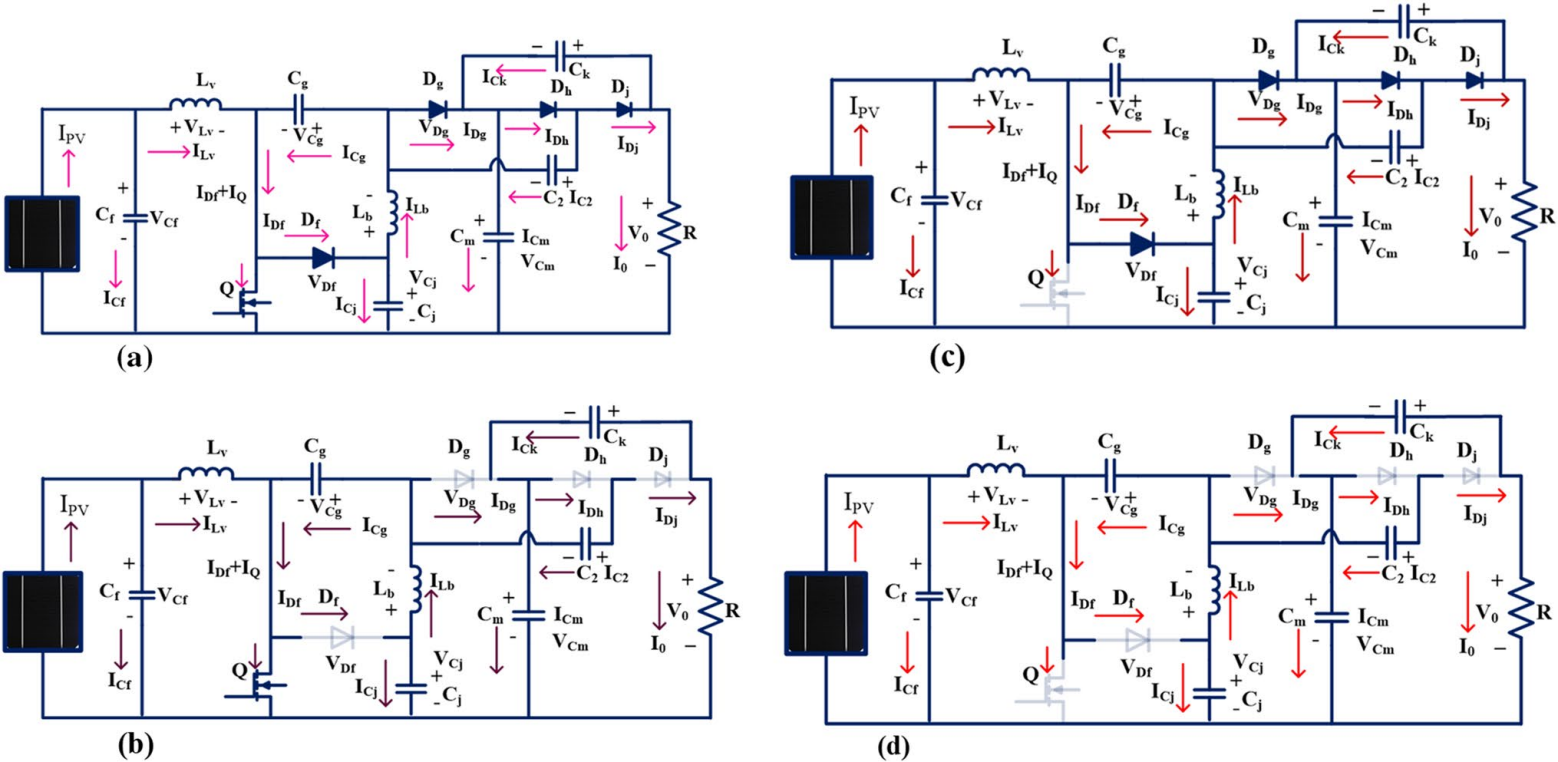
(**a**) Proposed wide voltage gain DC-DC power converter, (**b**) The first working stage of the proposed converter, (**c**) 2nd working stage of the proposed converter, (**d**) Converter works in blocking state of operation.

The solar power network per unit power generation price is higher, and its utilization factor is also less. So, the researchers refer to the power electronics converters for optimizing the power generation cost of the sunlight system^29^. From the previously published literature articles, the isolated-based power converters needed high development costs, more size, high complexity in installation, plus high ripples in the power supply. Also, it needed additional rectifier devices for enhancing the voltage conversion ratio of the isolated converter circuits^30^. So, the non-isolated power converters play a predominant role in the present solar systems. Here, in this manuscript, a new non-isolated universal supply voltage power converter is introduced to increase the efficiency of the sunlight power generation system. The introduced converter structure is mentioned in the Fig. 10a. From Fig. 10a, the converter structure is designed by L_v_, and L_b_, and five capacitors C_f_, C_g_, C_j_, C_m_, C_2_, plus C_k_ selecting the two identical inductors which are named as. Here, the MOSFET device is selected for operating the overall system at a high-frequency range. The working states of the universal supply voltage converter is explained in Table 1.

**Table 1:** Working states of the high step-up boost converter.

Components	1st State (CCM & DCM)	2nd Stat (CCM & DCM)	3rd State (DCM)
Q	Functioning	Not functioning	Not functioning
D_f_	Not functioning	Functioning	Not functioning
D_g_	Not functioning	Functioning	Not functioning
D_h_	Not functioning	Functioning	Not functioning
D_j_	Not functioning	Functioning	Not functioning

### Functioning state of the converter-1

From Fig. 10b, when the MOSFET starts functioning, the selected elements L_v_, plus L_b_ start collecting the source energy which is represented as V_Lv_, plus V_Lb_. The currents flowing through the inductive elements rise from origin to I_Lv_, plus I_Lb_ respectively. The available voltages of inductors L_v_, plus L_b_ are V_Lv_, and V_Lb_. Here, the current passing through the switch is I_Q_ and its corresponding voltage is V_Q_. Here, the elements D_g_, D_h_, C_j_, C_2_, C_k_, plus L_b_ act as the filter circuit for suppressing the fluctuations of input supply power. The capacitor's currents are named I_Cf_, I_Cg_, I_Cj_, I_Cm_, and I_Ck_ and its related voltages are V_Cf_, V_Cg_, V_Cj_, V_Cm_, and V_Ck_. In this converter structure, some of the capacitive elements consume the energy, and the reaming capacitors deliver the energy which are named I_Cf_carg_, I_Cg_carg_ I_Cj_carg_, I_Cm_carg_, I_Cm_carg_, I_Ck_carg_, I_Cf_drgn_, I_Cg_drgn_ I_Cj_drgn_, I_Cm_drgn_, I_Cm_drgn_, and I_Ck_drgn_.

Similarly, the capacitive elements charge, plus discharge voltage parameters are represented as V_Cf_carg_, V_Cg_carg_ V_Cj_carg_, V_Cm_carg_, V_Cm_carg_, V_Ck_carg_, V_Cf_drgn_, V_Cg_drgn_ V_Cj_drgn_, V_Cm_drgn_, V_Cm_drgn_, and V_Ck_drgn_. For identifying the suitable duty value of the converter, there are 3 assumptions utilized in the converter structure which are the 1^st^ one is all the capacitors, plus inductors must and should have higher-rated values. The 2^nd^ one is that all the switches are ideal, and the 3^rd^ one is all the devices, and electrical elements' internal resistances are zero. In this converter functioning stage, the following -diodes D_f_, D_g_, plus D_h_ are in a completely blocking stage. Here, the supplied voltage V_gs_ is selected high value in order to converter circuit in DCM, plus CCM of operations. The converter source side elements C_f_, and C_g_ consume the energy, and the elements C_m_, C_2_, C_j,_ and C_k_ give the energy to the load resistor. From Fig. 10b, the inductor voltages are derived as,

17 $$\left\{\begin{array}{c}{{\text{V}}}_{{\text{Lv}}}={{\text{V}}}_{{\text{FC}}}\\ {{\text{V}}}_{{\text{Lb}}}={{\text{V}}}_{{\text{Cj}}\_{\text{drgn}}}-{{\text{V}}}_{{\text{Cg}}\_{\text{carg}}}\end{array}\right.$$

18 $$\left\{\begin{array}{c}{{\text{I}}}_{{\text{Cg}}\_{\text{carg}}}={{\text{I}}}_{{\text{Q}}}-{{\text{I}}}_{{\text{Lv}}}\\ {{\text{I}}}_{{\text{Cg}}\_{\text{carg}}}-{{\text{I}}}_{{\text{C}}2\_{\text{carg}}}={{\text{I}}}_{{\text{Lb}}}=-{{\text{I}}}_{{\text{Cj}}\_{\text{drgn}}}\\ {{\text{I}}}_{{\text{Ck}}\_{\text{drgn}}}={{\text{I}}}_{{\text{C}}2\_{\text{carg}}}+{{\text{I}}}_{{\text{Cm}}\_{\text{drgn}}}=-{{\text{I}}}_{0}\end{array}\right.$$

### Functioning state of the converter-2

Here, the MOSFET device gate-supplied voltage is reduced to a very low level by using the gate driver network then the operating switch (Q) moves in the blocking state, and the diodes (D_f_, D_g_, plus D_h_) go into a functioning state which is explained in Fig. 10c. From Fig. 10c, the diode voltages (V_Cf_, V_Cg_, V_Cj_, V_Cm_, and V_Ck_) are zero, and the switch voltage is equal to V_Q_. In this state also, the converter circuit tries to function in DCM and CCM operations. Here, the capacitive elements C_f_, and C_g_ deliver the energy to the load resistor via conducting diodes. The load-side capacitive components (C_m_, C_2_, C_j,_ and C_k_) try to consume the power to stabilize the consumer voltage. Here, Kirchhoff's Voltage Law, plus Kirchhoff's Current Laws are applied to the proposed converter structure to determine the currents flowing through the circuit.

19 $$\left\{\begin{array}{c}{{\text{I}}}_{{\text{Cg}}\_{\text{drgn}}}={{\text{I}}}_{{\text{Df}}}-{{\text{I}}}_{{\text{Lv}}}\\ {{\text{I}}}_{{\text{C}}2\_{\text{drgn}}}={{\text{I}}}_{{\text{Dg}}}+{{\text{I}}}_{{\text{Cg}}\_{\text{drgn}}}-{{\text{I}}}_{{\text{Lv}}}\\ {{\text{I}}}_{{\text{Cj}}\_{\text{char}}}={{\text{I}}}_{{\text{Lv}}}-{{\text{I}}}_{{\text{Df}}}\\ {{\text{I}}}_{{\text{Ck}}\_{\text{chrg}}}={{\text{I}}}_{{\text{Cm}}\_{\text{chrg}}}-{{\text{I}}}_{{\text{Dg}}}\end{array}\right.$$

### Functioning state of the converter-3

**Figure 11:**
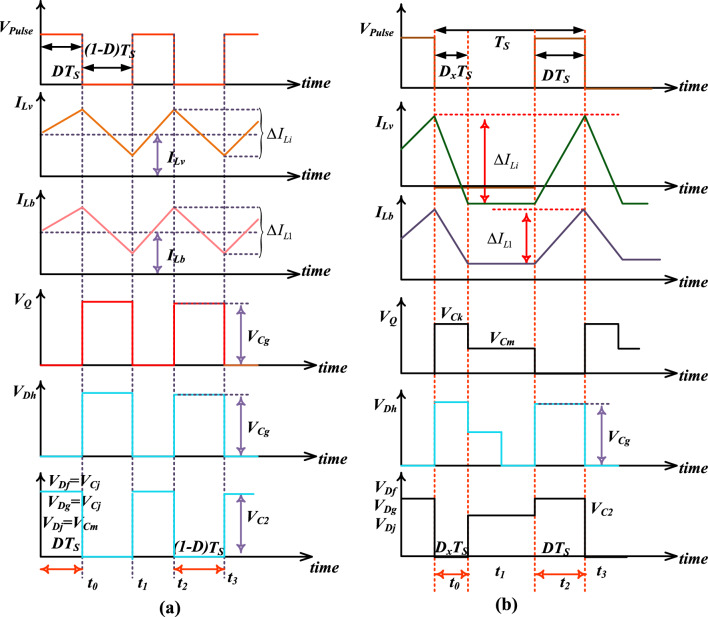
Introduced converter structure works under, (**a**). Continuous conduction stage, plus (**b**). Discontinuity conduction state.

From Fig. 10d, in this stage, the introduced converter structure works in the discontinuous power supply mode of operation. Here, all the semiconductor devices stop working, and the energy storage elements supply the energy to the resistive load. From Fig. 11a,b, the average voltage that appears across the inductor is ^‘^0^’^.

20 $${{\text{V}}}_{{\text{Lv}}\_{\text{Minimum}}}={{\text{V}}}_{{\text{Lb}}\_{\text{Minimum}}}=0$$

21 $${{\text{I}}}_{{\text{Lv}}\_{\text{Minimum}}}+{{\text{I}}}_{{\text{Lb}}\_{\text{Minimum}}}=0$$

The voltage conversion of the introduced converter is evaluated by selecting the Fig. 10a,b. The steady-state investigation of the proposed converter has been done by focusing on the inductor voltages. From Fig. 11a, the converter switch starts functioning then the inductors start consuming the power. Otherwise, the selected inductors give the energy to the capacitors as shown in Fig. 11b.

22 $${{\text{V}}}_{{\text{Cg}}}=\frac{{\text{D}}}{(1-{\text{D}})}*{{\text{V}}}_{{\text{FC}}}$$

23 $${{\text{V}}}_{{\text{C}}2}={{\text{V}}}_{{\text{Cj}}}={{\text{V}}}_{{\text{Ck}}}=\frac{1}{(1-{\text{D}})}*{{\text{V}}}_{{\text{FC}}}$$

24 $${{\text{V}}}_{{\text{Cm}}}=\frac{1+{\text{D}}}{(1-{\text{D}})}*{{\text{V}}}_{{\text{FC}}}$$

25 $${{\text{V}}}_{0}=\frac{2+{\text{D}}}{(1-{\text{D}})}*{{\text{V}}}_{{\text{FC}}}$$

26 $${{\text{Gan}}}_{{\text{CCM}}}=\frac{{{\text{V}}}_{0}}{{{\text{V}}}_{{\text{PV}}}}=\frac{2+{\text{D}}}{(1-{\text{D}})}$$

27 $$\left\{\begin{array}{c}{{\text{V}}}_{{\text{Q}}}={{\text{V}}}_{{\text{D}}}=\frac{1}{(1-{\text{D}})}*{{\text{V}}}_{{\text{FC}}}\\ {{\text{V}}}_{{\text{D}}}={{\text{V}}}_{{\text{Df}}}={{\text{V}}}_{{\text{Dg}}}={{\text{V}}}_{{\text{Dh}}}={{\text{V}}}_{{\text{Dj}}}\end{array}\right.$$

28 $${{\text{V}}}_{{\text{S}}}={{\text{V}}}_{{\text{D}}}=\frac{2+{{\text{Gan}}}_{{\text{CCM}}}}{3*{{\text{Gan}}}_{{\text{CCM}}}}*{{\text{V}}}_{0}$$

29 $${{\text{I}}}_{{\text{Lv}}}={{\text{I}}}_{{\text{Lb}}}={{\text{I}}}_{0}$$

30 $${{\text{I}}}_{{\text{Lv}}}=\frac{2+{\text{D}}}{1-{\text{D}}}*{{\text{I}}}_{0}={{\text{Gan}}}_{{\text{CCM}}}*{{\text{I}}}_{0}$$

### Comprehensive investigation of introduced converter circuit

Basically, any power DC-DC converter is utilized for sunlight power generation systems based on the power conduction losses of the entire system, space required for installation, handling capability, plus design flexibility. The isolated converter circuit involves more rectifiers and other devices for improving the voltage stability of the system. The general boost converter is replaced in place of the isolated converters to optimize the size of the renewable energy network. The general converter takes a high value of duty for enhancing the functioning efficiency of the hybrid renewable power distribution network. However, these converter energy losses are very excessive at the operating high-duty value of the switch. So, an inductor-coupled resonant converter is utilized in^39^ for the solar-powered electric vehicle system. This converter takes four inductors for filtering the fluctuations of wind/PV power system.

**Figure 12:**
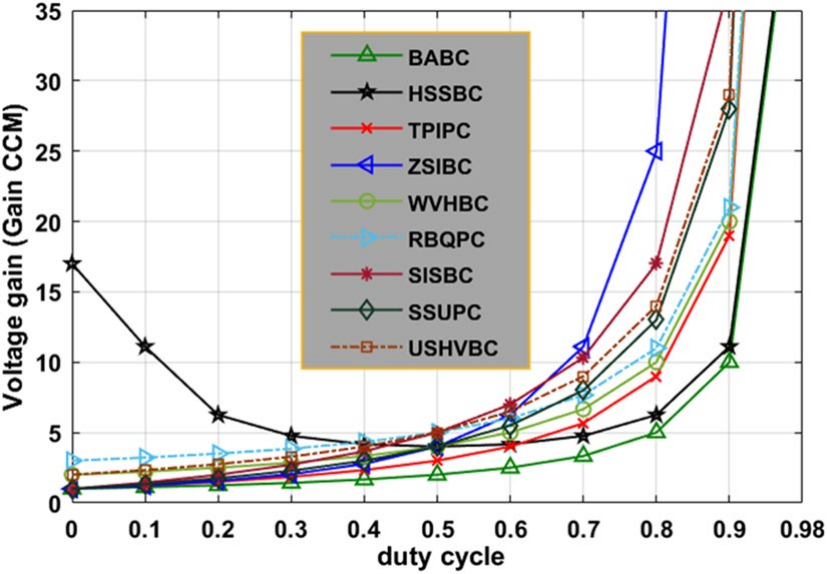
Effect of voltage conversion ratio with respect to duty variation.

**Figure 13:**
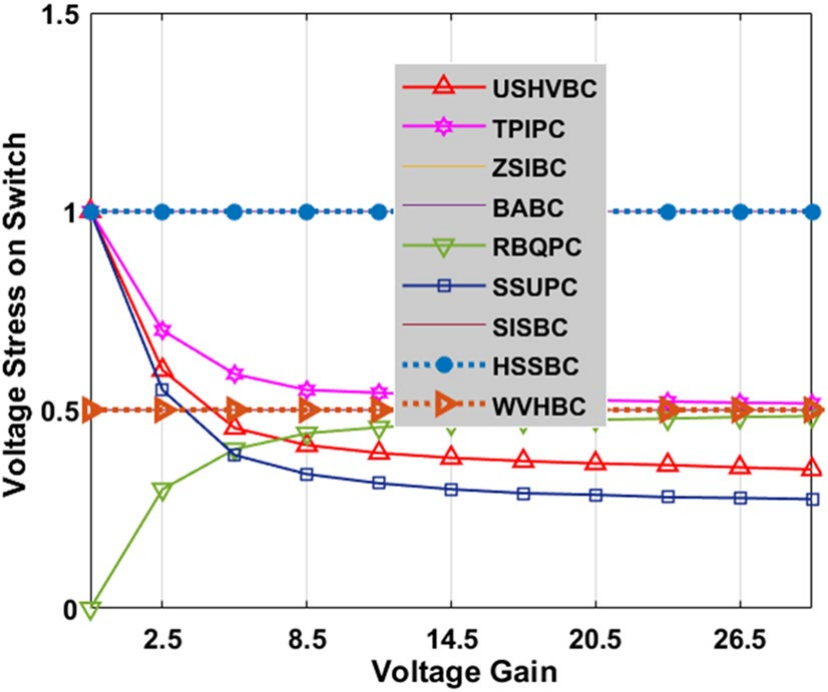
Effect of voltage conversion ratio concerning voltage stress on switches.

**Figure 14:**
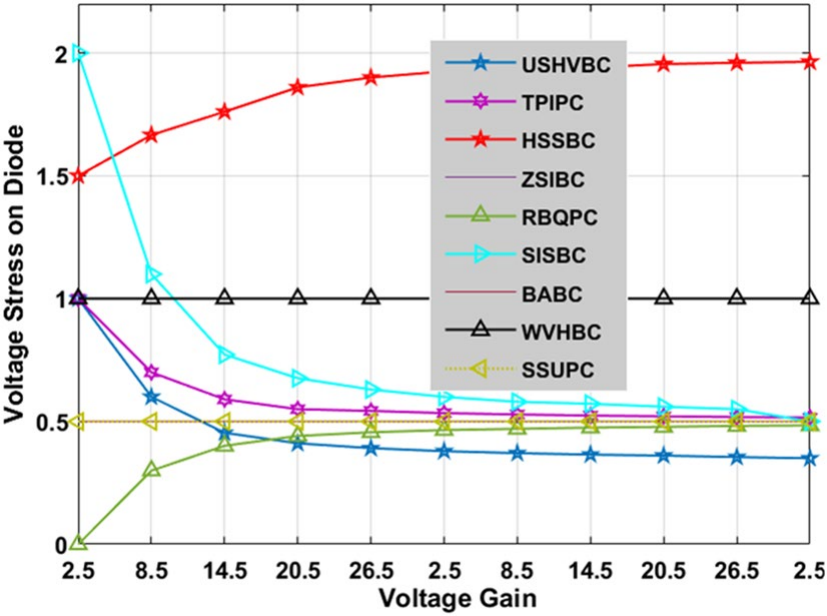
Effect of voltage conversion ratio with respect to voltage stress on diodes.

There are various types of interleaved converter circuits are investigated in^40^ for high-power automotive systems to give continuous electrical energy to the induction motors thereby running the electric vehicle with a uniform speed. The Z-source circuit-based high voltage gain converter is replaced with the interleaved circuit to reduce the implantation cost of the water pumping system. The dual input source and single output power converter are designed by utilizing the four switches, plus two diodes for moderate power rating centrifugal power systems to reduce the load current of the local loads. The investigation of the introduced converter along with the other converter circuits is explained in Table 2. The fluctuations of converters' voltage conversion ratios with associated the duty cycle variation are given in Fig. 12. From Fig. 12, the introduced converter voltage conversion ratio is kept on improving by improving the duty value of the converter. Similarly, how the voltage gain affects the voltage stress on switches, and diodes is illustrated in Fig. 13, and Fig. 14.

**Table 2:** Analysis of the introduced converter along with the previously existing converter circuits.

Circuit	Voltage Conversion	Devices Required	Ground	Energy storage elements	Type of source	MOSFET Voltage Stress	Diode Voltage Stress
BABC^31^	$\frac{1}{1-{\text{D}}}$	1 MOSFET 1 Diode	No need	1 Inductors 1 Capacitors	Uniform	1	1
SSUPC^32^	$\frac{1+2{\text{D}}}{1-{\text{D}}}$	1 MOSFET 3 Diodes	Need	3 Inductors 5 Capacitors	Fluctuated	$\frac{{{\text{Gan}}}_{{\text{CCM}}}+2}{3{{\text{Gan}}}_{{\text{CCM}}}}$	$\frac{{{\text{Gan}}}_{{\text{CCM}}}+2}{3{{\text{Gan}}}_{{\text{CCM}}}}$
RBQPC^33^	$\frac{3-{\text{D}}}{1-{\text{D}}}$	1 MOSFET 4 Diodes	No need	2 Inductors 5 Capacitors	Fluctuated	$\frac{{{\text{Gan}}}_{{\text{CCM}}}-1}{2{{\text{Gan}}}_{{\text{CCM}}}}$	$\frac{{{\text{Gan}}}_{{\text{CCM}}}-1}{2{{\text{Gan}}}_{{\text{CCM}}}}$
SISBC^34^	$\frac{1+3{\text{D}}}{1-{\text{D}}}$	2 MOSFETs 2 Diodes	Need	3 Inductors 5 Capacitors	Uniform	$\frac{1}{2}$	$\frac{1}{2}$
HSSBC^35^	$\frac{1}{{\text{D}}(1-{\text{D}})}$	2 MOSFETs 3 Diodes	Need	2 Inductors 2 Capacitors	Fluctuated	$\frac{1}{2}+\sqrt{\frac{1}{4}-\frac{1}{{{\text{Gan}}}_{{\text{CCM}}}}}$	$\frac{3}{2}+\sqrt{\frac{1}{4}-\frac{1}{{{\text{Gan}}}_{{\text{CCM}}}}}$
TPIPC^36^	$\frac{1+{\text{D}}}{1-{\text{D}}}$	1 MOSFET 4 Diodes	No Need	2 Inductors 3 Capacitors	Uniform	$\frac{1+{{\text{Gan}}}_{{\text{CCM}}}}{2{{\text{Gan}}}_{{\text{CCM}}}}$	$\frac{1+{{\text{Gan}}}_{{\text{CCM}}}}{2{{\text{Gan}}}_{{\text{CCM}}}}$
ZSIBC^37^	$\frac{1}{(1-{\text{D}})(1+{\text{D}})}$	1 MOSFET 3 Diodes	Need	2 Inductors 2 Capacitors	Fluctuated	1	1
USHVBC	$\frac{2+{\text{D}}}{1-{\text{D}}}$	1 MOSFET 4 Diodes	Need	2 Inductors 4 Capacitors	Uniform	$\frac{3+{{\text{Gan}}}_{{\text{CCM}}}}{4{{\text{Gan}}}_{{\text{CCM}}}}$	$\frac{3+{{\text{Gan}}}_{{\text{CCM}}}}{4{{\text{Gan}}}_{{\text{CCM}}}}$
WVHBC^38^	$\frac{2}{1-{\text{D}}}$	2 MOSFETs 2 Diodes	Need	2 Inductors 2 Capacitors	Fluctuated	$\frac{1}{2}$	$\frac{1}{2}$

### Components design and selection of the proposed converter

The converter design for the sunlight power generation system is a very important task because solar module efficiency depends on the inductors and capacitors' selection of the converter. Here, the MOSFET switch is selected based on Eq. (25) and Eq. (28). This switch operates under a continuous conduction stage with acceptable voltage stress on it. Similarly, the diodes are operated in a safe operating area with low-level voltage stress on it. Here, the inductors (L_v_, plus L_b_) are designed by using Eq. (31), and the inductor's saturation current should be higher than the maximum current flowing through it. Also, the working inductor ripples (ΔL_v_, plus ΔL_b_) should have a minimum value then only the introduced converter supplies constant voltage to the consumers.

31 $$\left\{\begin{array}{c}{{\text{L}}}_{{\text{V}}}\ge \frac{{\text{D}}*{{\text{V}}}_{{\text{FC}}}}{{{\text{F}}}_{{\text{s}}}*{\Delta {\text{I}}}_{{\text{Lv}}}}\\ {{\text{L}}}_{{\text{b}}}\ge \frac{{\text{D}}*{{\text{V}}}_{{\text{FC}}}}{{{\text{F}}}_{{\text{s}}}*{\Delta {\text{I}}}_{{\text{Lb}}}}\end{array}\right.$$

32 $$\left\{\begin{array}{c}\begin{array}{c}{{\text{C}}}_{{\text{f}}}\ge \frac{{\text{D}}*{2{\text{I}}}_{0}}{{{\text{F}}}_{{\text{s}}}*{\Delta {\text{V}}}_{{\text{Cf}}}}\\ {{\text{C}}}_{{\text{g}}}\ge \frac{{\text{D}}*{2{\text{I}}}_{0}}{{{\text{F}}}_{{\text{s}}}*{\Delta {\text{V}}}_{{\text{Cg}}}}\end{array}\\ {{\text{C}}}_{{\text{j}}}\ge \frac{{\text{D}}*{2{\text{I}}}_{0}}{{{\text{F}}}_{{\text{s}}}*{\Delta {\text{V}}}_{{\text{Cj}}}}\\ \begin{array}{c}{{\text{C}}}_{2}\ge \frac{{\text{D}}*{2{\text{I}}}_{0}}{{{\text{F}}}_{{\text{s}}}*{\Delta {\text{V}}}_{{\text{C}}2}}\\ {{\text{C}}}_{{\text{k}}}\ge \frac{{\text{D}}*{2{\text{I}}}_{0}}{{{\text{F}}}_{{\text{s}}}*{\Delta {\text{V}}}_{{\text{Ck}}}}\end{array}\end{array}\right.$$

### Ethical approval

This paper does not contain any studies with human participants or animals performed by any of the authors.

## Simulation results

The proposed advanced adaptive genetic algorithm optimized RBFN controller is developed by utilizing the MATLAB/Simulink software. The working structure of the hybrid RBFN controller is explained in Fig. 2. Here, the hybrid RBFN controls the input equivalent solar PV impedance by controlling the duty cycle of the universal supply voltage DC-DC converter. The selected parameters for this MPPT method are five which are illustrated in Table 3. From Table.3, the selected rated power of each solar module is 254.82W, and its associated voltage is equal to 30.3 V. Here, there are three various diodes are utilized for designing the sunlight system. So, the total number of ideality factors of the diodes is three which are equal to 0.84, 0.94, and 1 respectively.

**Table 3:** Design parameters of the sunlight network.

Parameters	Value
Rated utilized power of solar network (P_mpp_)	254.82W
The rated utilized voltage of Solar (V_mpp_)	30.3 V
Open-circuited condition PV voltage (V_oc_)	37.5 V
Rated utilized current of solar network (I_mpp_)	8.41A
Short-circuited PV current (I_sc_)	8.85A
Utilized series-placed strings	1
Utilized parallel-placed strings	1
Resistance of the series cell (R_y_)	0.458 Ω
Resistance of the parallel cell (R_k_)	877.781 Ω

The 3-diode model sunlight network functioning efficiency is a little high when associated with the other solar networks. However, this 3-diode solar module supplying power is low which is enhanced by applying the wide voltage gain uniform supply voltage DC-DC converter.

### Analysis of sunlight power system at 1000W/m^2^

The utilized converter inductor (L_v_ and L_b_) and capacitor (C_f_, C_g_, C_j_, C_m_, C_2_, plus C_k_) values and their associated internal resistances are equal to 250µH, 220µF, r_L_ = 0.01Ω, and rc = 0.1Ω respectively. Here, the overall PV module rating is 764W and it is studied by interfacing the various ANN-based hybrid MPPT controllers which are P&O-ANN, HC with ANN, and GA with P&O controllers. Here, at the initial testing stage, the 3 solar modules' incident sunlight insolation is uniform and its generated output power is also uniform because it has a single functioning point and the incident irradiations on the three PV modules are equal to 1000W/m^2^.

**Figure 15:**
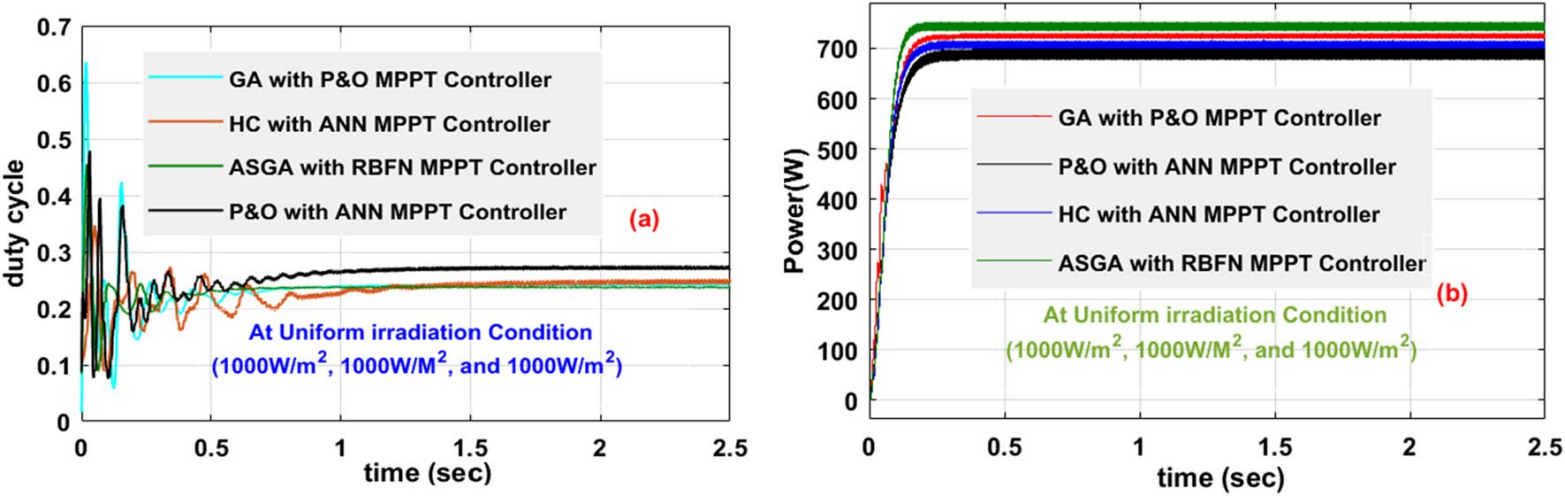
The introduced USHVBC power converter, (**a**). Duty signal, plus (**b**). Converter output power at 1000W/m^2^.

At 1000W/m^2^ sunlight insolation, the available power from the sunlight system by integrating the adaptive proposed MPPT circuit-based USHVBC is 752.96W, and its functioning efficiency is 98.84%. The rising, plus settling time duration of the USHVBC output voltage by interfacing the P&O-ANN, HC with ANN, GA with P&O, plus ASGA-RBFN controllers under uniform sunlight conditions are 0.83Sec, 0.80Sec, 0.66Sec. 0.48Sec, 1.31Sec, 1.22Sec, 1.1Sec, plus 0.82Sec respectively. Under constant sunlight irradiations, the maximum available voltage, plus efficiency of the introduced system by applying the P&O-ANN, HC with ANN, plus GA with P&O controller are 219.36 V, 225.11 V, 225.99 V, 93.81%, 94.91%, plus 97.01%. The detailed results investigation of the USHVBC-fed GA-RBFN system are explained in Table 4. From Table.4, the voltage fluctuations of the converter by using the P&O-ANN, and HC with ANN controllers are more when equated with the GA with P&O, and ASGA-RBFN controllers. The fluctuations of the USHVBC operating duty cycle and its corresponding available power are shown in Fig. 15a, plus Fig. 15b.

**Table 4:** Detailed investigation of Adaptive GA-RBFN Controller at Various Sunlight Conditions

Controller	USHVBC Voltage (V_0_)	USHVBC Power (P_0_)	Efficiency (η%)	Fluctuations in USHVBC Voltage	Converter Voltage Rise time	Converter Voltage Settling time	MPP Tracking speed	Accuracy of the MPPT Method
Investigation of the USHVBC fed Sunlight System at 1000W/m^2^								
P&O-ANN	219.36 V	707.29W	93.81%	More	0.83Sec	1.31Sec	Low	Low
HC-ANN	225.11 V	729.18W	94.91%	More	0.80Sec	1.22Sec	Low	Low
GA-P&O	225.99 V	735.44W	97.01%	Medium	0.66Sec	1.1Sec	Medium	Low
ASGA-RBFN	239.89 V	752.96W	98.84%	Low	0.48Sec	0.82Sec	Fast	More
Investigation of the USHVBC fed Sunlight System at 1000W/m^2^, 894W/m^2^, and 694W/m^2^								
P&O-ANN	174.17 V	565.19W	92.01%	More	0.98Sec	1.42Sec	Low	Low
HC-ANN	176.02 V	584.10W	93.99%	More	0.96Sec	1.81Sec	Low	Low
GA-P&O	180.11 V	586.92W	96.11%	Medium	0.81Sec	0.99Sec	Medium	Low
ASGA-RBFN	181.99 V	600.76W	97.77%	Low	0.51Sec	0.93Sec	Fast	More
Investigation of the USHVBC fed Sunlight System at 1000W/m^2^, 694W/m^2^, and 594W/m^2^								
P&O-ANN	170.22 V	492.22W	90.55%	More	1.01Sec	1.44Sec	Low	Low
HC-ANN	171.94 V	504.34W	92.92%	More	1.28Sec	1.91Sec	Low	Low
GA-P&O	172.67 V	513.82W	93.89%	Medium	0.96Sec	2.21Sec	Medium	Low
ASGA-RBFN	173.18 V	515.12W	96.19%	Low	0.53Sec	1.27Sec	Fast	More

### Analysis of sunlight power system at 1000W/m^2^, 894W/m^2^, and 694W/m^2^

**Figure 16:**
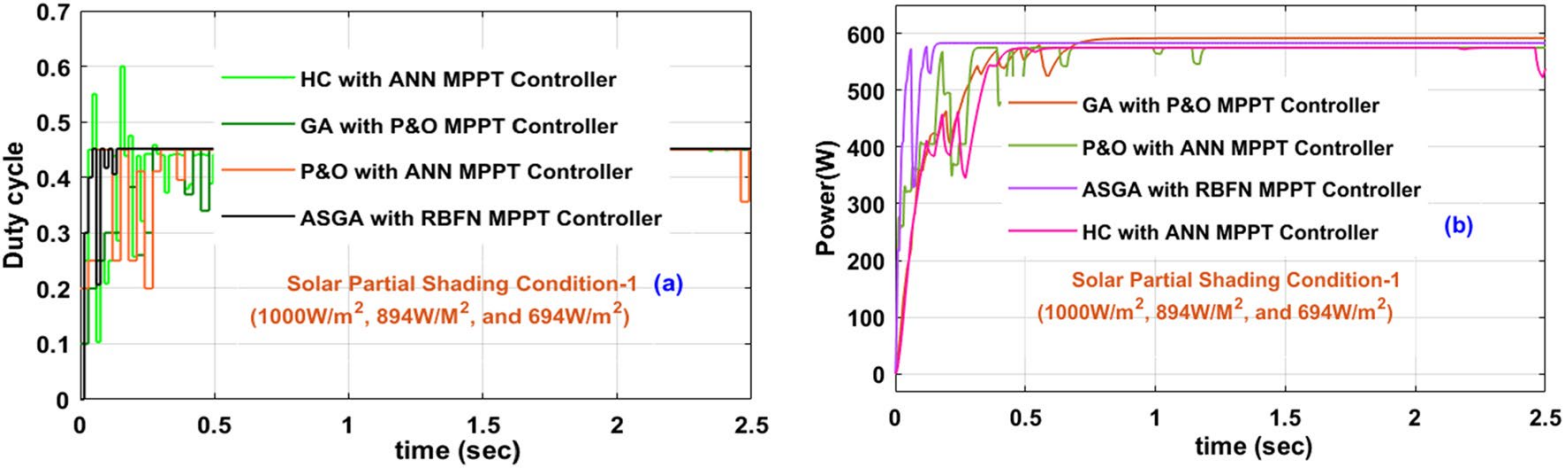
The introduced USHVBC power converter, (**a**). Duty signal, plus (**b**). Converter output power at 1000W/m^2^, 894W/m^2^, and 694W/m^2^.

Here, the sunlight irradiations vary from time to time because of the continuous variation of the sunlight intensity. From Fig. 16a, at 0 s, the sunlight irradiation value is 1000W/m^2^, and it changes to 894W/m^2^ at 0.83 s. Finally, it moves to 694W/m^2^. At this sudden change in sunlight conditions, the overall system power utilization is reduced as shown in Fig. 16b. At this first PSC, the available voltage, plus power of overall system, and its efficiency by combining the P&O-ANN, HC with ANN, GA with P&O, plus ASGA-RBFN controllers are 174.17 V, 565.19W, 92.01%, 176.02 V, 584.10W, 93.99%, 180.11 V, 586.92W, 96.11%, 181.99 V, 600.76W, plus 97.77%.

The P&O-ANN method creates heavy distortions in the overall system, and it identifies the MPP speed is also low for rapid variations of the sunlight intensity conditions. In addition, the accuracy of MPP tracking is low, and it does not give a constant voltage to the resistive load. The converter voltage settling and sudden rise timings from the utilization of all MPPT controllers are 1.42Sec, 1.81Sec, 0.99Sec, 0.93Sec, 0.98Sec, 0.96Sec, 0.81Sec, plus 0.51Sec respectively. Here, the introduced adaptive GA-RBFN method implementation is a little tough. However, it gives very low-level distortions, plus easy handling.

### Analysis of sunlight power system at 1000W/m^2^, 694W/m^2^, and 594W/m^2^

**Figure 17:**
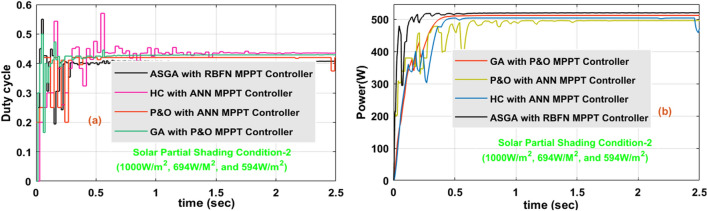
The introduced USHVBC power converter, (**a**). Duty signal, plus (**b**). Converter output power at 1000W/m^2^, 694W/m^2^, and 594W/m^2^.

In this 2nd shading condition of the sunlight system, the power supply to the consumer is still reduced when equated with the 1st shading condition because the incident irradiations on the solar PV module are progressively reduced. The USHVBC functioning duty signal by the utilization of various power point identifiers is illustrated in Fig. 17a, and its associated consumer power waveforms are given in Fig. 17b. At 1000W/m^2^, 694W/m^2^, and 594W/m^2^, from Fig. 17a,b the proposed converter duty signal, plus power at load terminals based on the various P&O-ANN, HC with ANN, GA with P&O, plus ASGA-RBFN MPPT controllers are 0.53, 0.52, 0.5, 0.47, 492.22W, 504.34W, 513.82W, plus 515.12W. Similarly, at PSC-2, the converter voltage rising, settling periods, and their efficiencies based on the various P&O-ANN, HC with ANN, GA with P&O, plus ASGA-RBFN MPPT methods are 1.01Sec, 1.28Sec, 0.96Sec, 0.53Sec, 1.44Sec, 1.91Sec, 2.21Sec, 1.27Sec, 90.55%, 92.92%, 93.89%, plus 96.19% respectively. The P&O-ANN design complexity, plus understanding levels are low. But it creates heating and distortion losses in the system. So, from the above results investigation, the introduced adaptive step GA-RBFN is best suitable for the shading conditions of the solar power supply networks. Also, this introduced converter solves the all-complex problems of the sunlight system very easily and needs moderate-level knowledge candidates to train the overall system under dynamic sunlight conditions.

## Experimental verification of the proposed USHVBC

**Figure 18:**
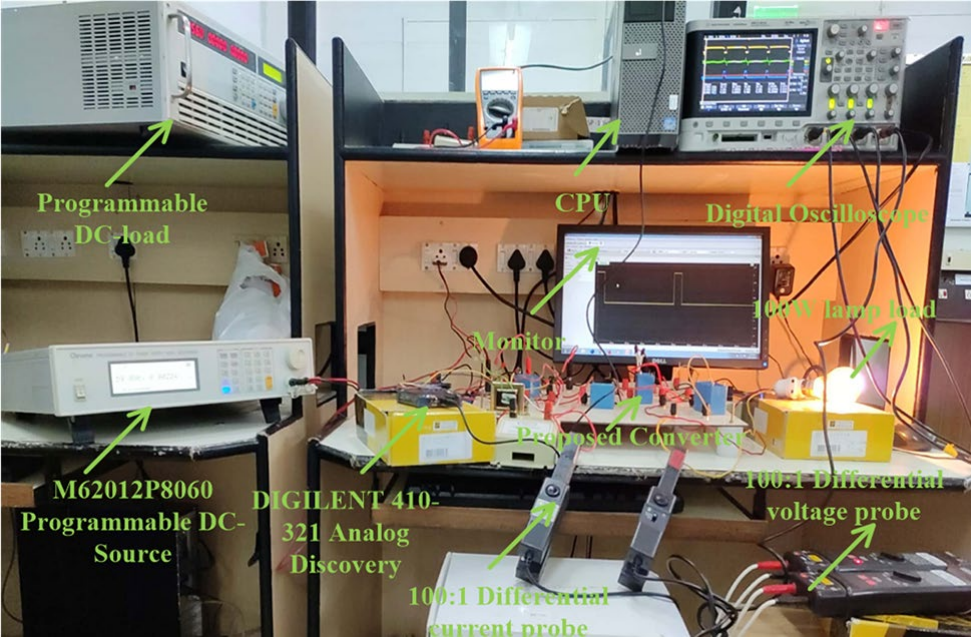
Overall testing of USHVBC converter circuit prototype.

The introduced converter design is already explained in Section “Development of Universal Input Supply DC-DC Converter”. In this proposed converter circuit, the element L_v_ filters the unwanted distortions in the supply voltage, and it tries to make the load voltage constant. Similarly, the elements C_f_, plus C_j_ are helpful for the load stabilization process at various dynamic sunlight conditions. The elements C_g_, C_j_, C_2_, D_g_, D_h_, L_b_ network are helpful for the enhancement of the voltage converter ratio of the introduced converter. The selected components for the design of the converter network and their respective values are given in Table 5. The implementation of the USHVBC circuit is represented in Fig. 18. From Fig. 18, here, the programmed DC supply device is utilized for the testing of the proposed circuit. Here, the 1000:1 IC variable current device, plus 1000:1 IC voltage devices are selected for the measurement of consumer load power. The 1-Φ transformer device is merged in between the local power supply, and MOSFET for supplying the rated voltage to the TLP-360 driver device.

**Table 5:** Utilized elements and their corresponding rating.

Device	Specification
Utilized switching device	IRF-840 MOSFET
Selected diodes for developing the circuit	IC-4N45
Functioning circuit frequency	20 kHz
Type driver & its voltage	IRF-350 & 5 V
Measuring device type & rating	1000:1 & 7 kV
Selected source device	DC source
Voltage available at capacitor C_f_ is (V_i_)	69.976 V
Voltage available at capacitor C_k_ is (V_0_)	122.002 V
Functioning time of Switch	5µsec
Blocking time of Switch	45µsec
C_f_, C_g_, C_j_, C_m_, C_2_, plus C_k_	250µF
L_v_ and L_b_	250µH
Tektronix DSO	TPS-2024B

**Figure 19:**
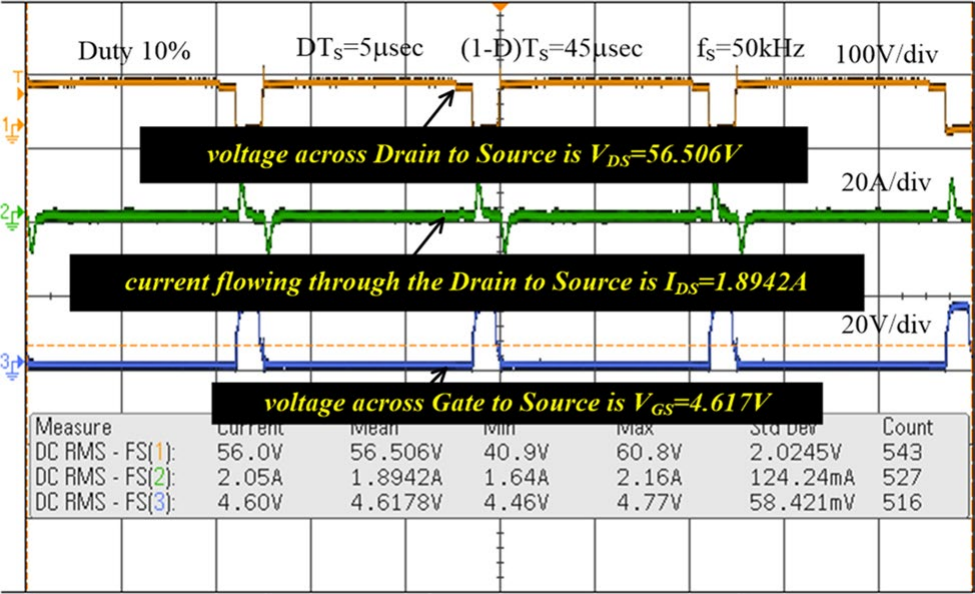
Functioning of MOSFET pulses, and their supply voltages.

The MOSFET switch is considered in this converter network because of its merits are high source impedance, high rated voltage controlling capability, required very low-level driving power, fast switching states, and ability to work up to 100A current rating. The TLP-350 is an 8-pin IC device and its input threshold current, and voltages are 8-10 mA, plus 5 V. The switching pulses to the MOSFET are supplied by utilizing the TLP-350 IC, and it helps the switch from the sudden changes of the short-circuited currents. In this driver circuit, the GAAIAs device is interfaced to indicate the sequential switching pulse generation. Here, the converter functioning frequency is 20 kHz, and its related converter switching pulses generation is represented in Fig. 19. From Fig. 19, the functioning duty value of the converter is 0.1. The supplied MOSFET drain-supply & gate-supply voltages are 56.5 V, plus 4.617 V respectively. The supplied MOSFET drain current is 1.894A. The analog discovery device is interfaced with the TLP-350 driver circuit for controlling the capacitor voltages.

**Figure 20:**
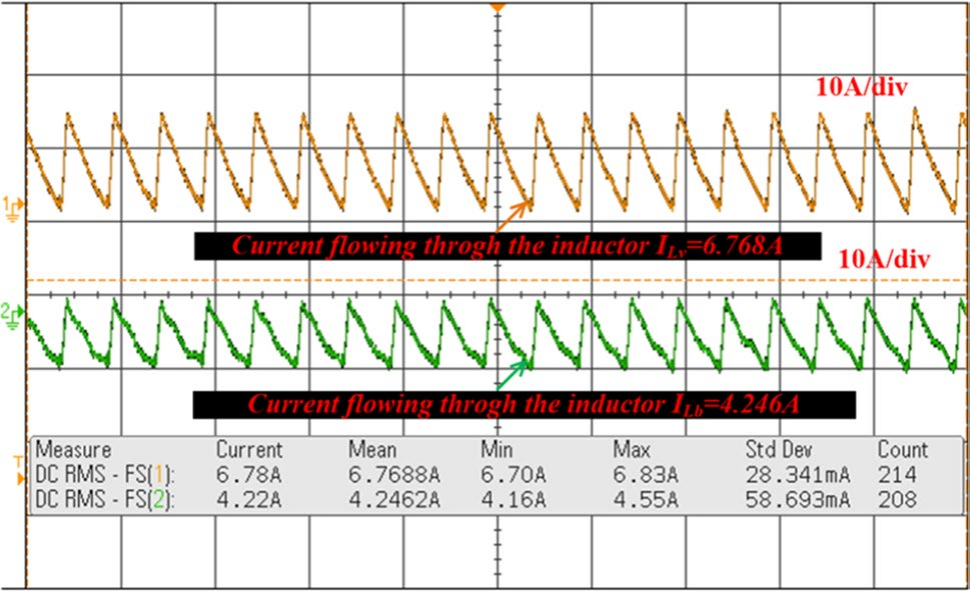
Supply currents of various inductors L_v_ and L_b_.

**Figure 21:**
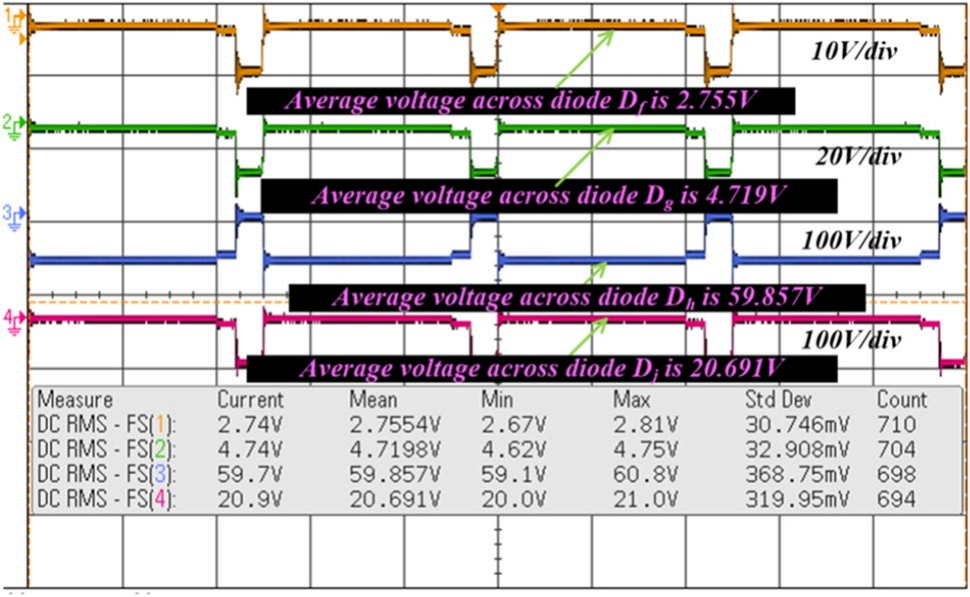
Available voltages of all the utilized diodes.

**Figure 22:**
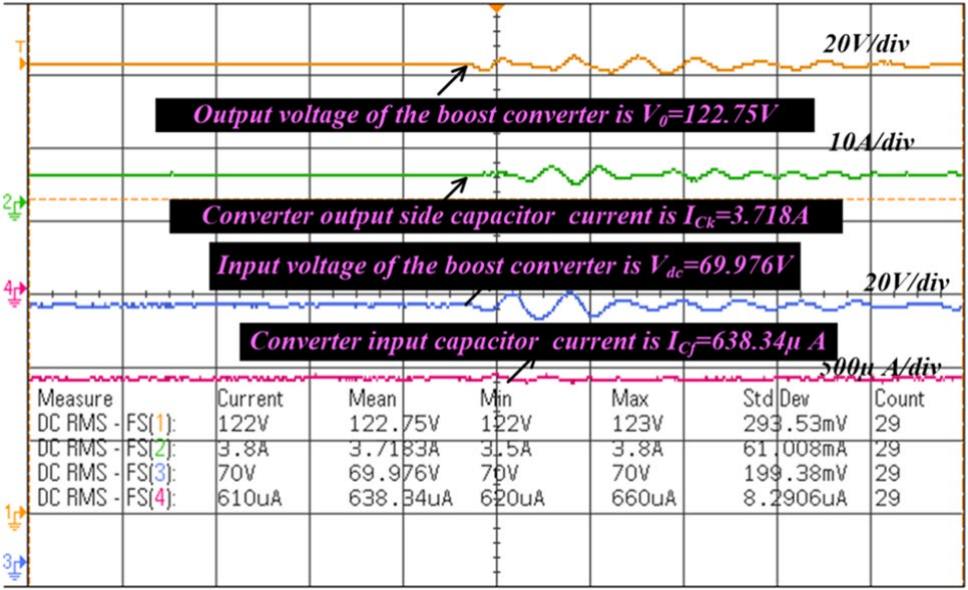
Available voltages of all the sources and loads.

**Figure 23:**
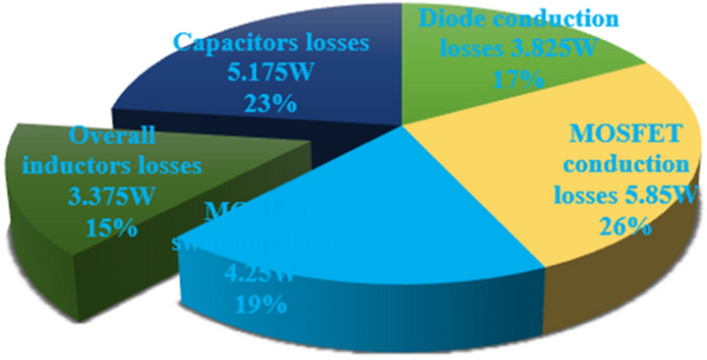
Introduced circuit distribution power losses.

**Figure 24:**
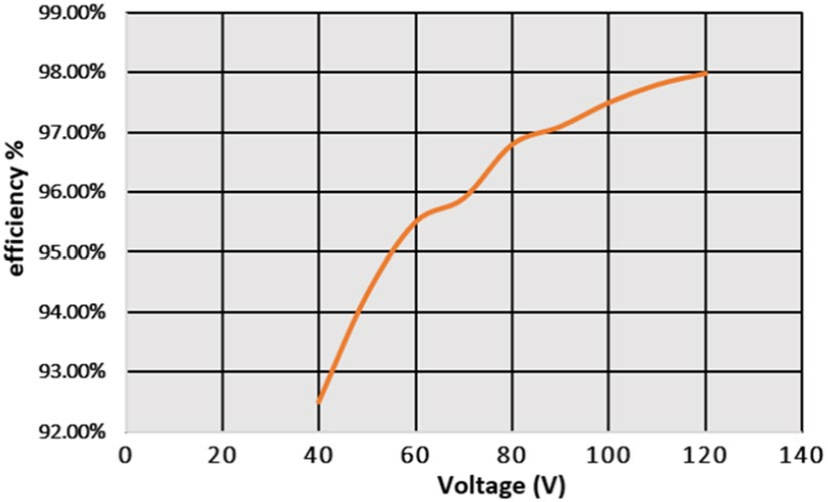
The performance efficiency of the USHVBC.

From Fig. 18, when the switch starts functioning, the utilized inductors (L_v_ and L_b_) collect the energy, and it delivers the energy to the filter circuit when the switch is blocked. The supply currents flowing over the inductors L_v_, plus L_b_ are equal to 6.76A, plus 4.2A respectively. The nature of current waveforms is illustrated in Fig. 20. The supplied voltages to the input terminals of the diodes D_f_, D_g_, D_h_, plus D_j_ are 2.75 V, 4.71 V, 59.85 V, plus 20.69 V as shown in Fig. 21. The utilized source signal to the converter is 69.97 V which is boosted to 122.7 V to meet peak load demand of the consumer, and the current available at the load is 3.71A which is very low when associated with the supply current. The voltage conversion of boost converter is given in Fig. 22. The formation of power losses in the converter circuit is given in Fig. 23, and its associated converter system efficiency curve is represented in Fig. 24. From Fig. 23, at 70 V supply, the entire proposed network power distribution loss is 22.5W, and the overall four diodes (D_f_, D_g_, D_h_, plus D_j_) power loss is 17% (3.825W) of the entire loss in the network. The switching, plus conduction loss of the network and its percentages in the total loss are 4.25W, 19%, 5.85W, plus 26% respectively. Finally, the capacitive, and inductive elements' power losses and their percentages in the overall loss are 5.715W, 23%, 3.375W, plus 15% respectively.

## Conclusion

The ASGA-RBFN controller-fed universal supply high voltage gain boost converter is implemented by utilizing the MATLAB/Simulink tool. Here, in the first objective, the ASGA-RBFN controller is proposed for identifying the working point of the solar PV. From the simulation results, this proposed MPPT controller extracts the peak power from the sunlight power generation system at various dynamic irradiation conditions. The merits of this MPPT controller are quick response, low fluctuations across the solar MPP, more accuracy, plus ease of handling. In the second objective, a universal supply voltage DC-DC converter is introduced for the shading condition of the sunlight system. Here, the introduced converter is tested with a programmable power source. From the converter tested results, the merits of this converter are very low-level voltage stress on switches, low power loss of the energy storage elements, high voltage conversion ratio, plus suitability for wide power range renewable energy systems.

## Data Availability

The datasets used and/or analyzed during the current study available from the corresponding author on reasonable request.

## List of symbols

PV: Photovoltaic
ASGAO: Adaptive Step Genetic Algorithm Optimized
RBFN: Radial Basis Functional Network
RES: Renewable Energy Sources
MPPT: Maximum Power Point Tracking
SEPIC: Single-ended primary-inductance converter
MOSFET: Metal Oxide Semiconductor Field Effect Transistor
P&O: Perturb & observe
ANN: Artificial Neural Network
GA: Genetic Algorithm
HC: Hill Climb
DCM: Discontinuous Conduction Mode
CCM: Continuous Conduction Mode
V_Cf_, V_Cg_, V_Cj_, V_Cm_, and V_Ck_: Capacitor voltages
BABC: Basic Available Boost Converter
SSUPC: Single Switch Universal Power Converter
RBQPC: Reverse Bidirectional Quadratic Power Converter
SISBC: Single Input Soft Switching Boost Converter
HSSBC: High voltage gains Single Switch Boost Converter
TPIPC: Triple Phase Interleaved Power Converter
ZSIBC: Z-source soft switching Interleaved Boost Converter
USHVBC: Universal Supply High Voltage Gain Boost Converter
${{\text{net}}}_{{\text{q}}}^{1}({\text{j}})$: The available net value of the 1^st^ layer
${{\text{I}}}_{{\text{Sx}}}$: PV supply current of the system
${{\text{I}}}_{{\text{or}}\_{\text{v}}}$, ${{\text{I}}}_{{\text{or}}\_{\text{b}}}$,${{\text{I}}}_{{\text{or}}\_{\text{m}}}$: Available reverse saturation currents of the didoes
${{\text{I}}}_{{\text{Dv}}}$, ${{\text{I}}}_{{\text{Db}}}$,${{\text{I}}}_{{\text{Dm}}}$: All three diode currents for the sunlight system
V_Lv_, and V_Lb_: Converter inductor voltages
